# “I was afraid they will be judging me and even deny me the service”: Experiences of denial and dissuasion during abortion care in Ethiopia

**DOI:** 10.3389/fgwh.2022.984386

**Published:** 2022-11-01

**Authors:** Chiara Bercu, Laura E. Jacobson, Ewenat Gebrehanna, Ana Maria Ramirez, Anna J. Katz, Sofía Filippa, Sarah E. Baum

**Affiliations:** ^1^ Ibis Reproductive Health, Oakland, CA, United States; ^2^ School of Public Health, Oregon Health and Science University-Portland State University (OHSU-PSU), Portland, OR, United States; ^3^ St. Paul’s Hospital Millennium Medical College, Addis Ababa, Ethiopia

**Keywords:** abortion, Ethioipa, client-provider interaction, abuse in healthcare, disrespect and abuse (D&A), abortion stigma

## Abstract

**Introduction:**

Disrespect and abuse are components of poor quality abortion care. This analysis aimed to understand negative experiences of care from perspectives of abortion clients in public and private facilities in Ethiopia.

**Study Design:**

We conducted 23 in-depth interviews with people who obtained abortion care in Addis Ababa, Ethiopia as well as Aksum and Mekele in Tigray State, Ethiopia. The interviews were coded using *a priori* and emergent codes and we conducted thematic analysis to understand negative interactions with providers from participant's perspectives.

**Results:**

Participants experienced denial of abortion services along their pathway to care and attempts by providers to dissuade them prior to providing an abortion. Underlying both the denial and the dissuasion were reports of disrespect and condemnation from providers. Participants described how providers doubted or forced them to justify their reasons for having an abortion, stigmatized them for seeking multiple abortions or later abortions, and ascribed misinformation about abortion safety. Despite reports of denial, dissuasion, and disrespect, abortion clients generally felt that providers had their best interest at heart and were grateful for having access to an abortion.

**Conclusions:**

Participants in Ethiopia experienced providers as gatekeepers to legal abortion services, facing disrespect and judgment at facilities where they sought care. Interventions aimed at increasing awareness of abortion laws such that clients understand their rights and values clarification interventions for providers could help reduce barriers to accessing care and improve the quality of abortion services.

## Introduction

Decriminalizing and liberalizing abortion is an important step towards improving the availability of safe services, however, changes in abortion law do not ensure access to high-quality services. Even in contexts where abortion is legal, people are denied services and face a myriad of challenges in accessing high-quality care (1). People may face poor quality care along their pathways to care or during their abortions, including negative, stigmatizing, and discriminatory interactions with providers. Denial of services and poor interpersonal care from providers may have negative effects on the health outcomes and emotional well-being of those who seek abortion care (2).

Denial of safe abortion care can lead to long, winding trajectories to care, emotional distress for abortion seekers, and unsafe abortion practices which are a significant contributor to maternal mortality (3, 4). Denying an abortion may also have long-term implications including negative socioeconomic repercussions for families and negative developmental effects on existing children (5). Previous studies have documented some of the reasons for denial of services including: clients' lack of knowledge of legal availability of services, seeking care later in pregnancy, providers’ perception that abortion had negative health implications, logistical challenges including long wait times and insufficient funds, and abortion stigma (6–10). Gender inequity and systemic oppression of women, and other people capable of pregnancy^1^ may leave them vulnerable as patients in medical settings with little choice but to acquiesce to the power of providers (11). Implementation and interpretation of abortion laws vary greatly and access to abortion services often depends on the personal beliefs of providers (12, 13). Confusion and uncertainty surrounding abortion laws make health providers powerful gatekeepers to abortion services (14–17) which contributes to denial of services and impacts the quality of care provided.

It is well documented that obstetric violence, including disrespectful and abusive care, from providers is relatively commonplace in experiences of institutional birth (18–23). While disrespect and abuse during abortion care is less documented, studies have highlighted the importance of interpersonal aspects of care (24–26) as well as the failure to provide person-centered care in many contexts (27). Providing person-centered care is an important aspect of a high-quality abortion. A person-centered abortion takes into consideration an individual's preferences, their culture, and the context around them, and it includes dignity, autonomy, privacy, communication, social support, and trust (28, 29). One study assessing abortion-related obstetric violence in Latin America reported that providers criminalized, discriminated against, and were even physically violent with clients (30). Understanding service denial and disrespect from the perspective of people having abortions is important to improve person-centered care and develop interventions that can increase access to high-quality abortion services (31). This analysis aims to understand negative experiences of care from perspectives of abortion clients in Ethiopia.

### Ethiopia context

In Ethiopia, legal access to abortion was expanded in 2005 to include cases of rape, incest, and fetal impairment, if the pregnant person had a physical or mental disability, or if they were under 18 years of age (32). Abortion in such cases is legally permitted up to 28 weeks gestation. An additional clause in the law stated that the pregnant person's word was sufficient evidence of rape or incest, and that “stated age” was all that was needed to authorize an age-based abortion (33). In theory, this would have shifted power into the hands of abortion seekers and away from providers in determining eligibility for services. However, despite this expansion of rights in the country, abortion remains in the criminal code and is punishable by imprisonment (34), creating confusion and ambiguity around the law for providers and patients alike (14, 16, 35). Additionally, abortion service organizations have largely avoided sharing information on legal abortion in order to avoid politicizing their work and provoking anti-abortion sentiment, which has resulted in limited advocacy and access to information on safe abortion options (12). The most recent data available on abortions rates in Ethiopia are from 2014, that year it was estimated that 13% of unintended pregnancies ended in abortion or an estimated 620,300 abortions (36). The majority of these abortions were provided by private or NGO facilities, however, a large number of public health facilities and public hospitals also provided care and many more are capable of providing abortion and post-abortion care services (36). This analysis offers a deeper understanding of abortion experiences in private and public health facilities in Ethiopia with a focus on exploring negative client-provider interactions that may be impacting the quality of abortion services.

## Methods

In this study, we analyzed semi-structured in-depth interviews with people who obtained an abortion in either Addis Ababa or Aksum and Mek'ele in Tigray State (referred to as Tigray going forward), Ethiopia. These interviews were part of a larger study conducted in four countries with the goal of gaining a deeper understanding of people's experiences with abortion services and their perceptions of quality of care. For the purpose of this manuscript, we conducted a sub-analysis of interviews in Ethiopia with the aim of understanding negative experiences of abortion care from client's perspectives.

Three authors of this paper (SB, CB & AMR) collaborated with EG, an experienced qualitative researcher and a faculty member at St. Paul's Hospital Millennium Medical College (SPHMMC), school of public health to hire a local research team. EG served as the Study Coordinator and conducted interviews in Addis Ababa along with another trained qualitative researcher. Two additional qualitative researchers based at the University of Mekele were contracted to conduct data collection in Tigray region to ensure both linguistic and cultural considerations were taken into account for participants in that region. Two authors of this paper (CB & AMR) conducted trainings on ethical research, recruitment and qualitative interviewing techniques with the research team in-person in November 2018 and EG conducted a refresher training with data collectors in Tigray region in January 2019.

Participants were recruited through both private clinics in Addis Ababa, Ethiopia as well as public health facilities in Tigray. Participants who had obtained their abortions at private reproductive health clinics in Addis Ababa were recruited through a call center which provided clients with a referral to an affiliated clinic. The Call Center Coordinator called participants after they had completed their abortion to enquire on their interest in participating in this study. If they were interested in participating, their contact information was provided to EG to schedule a time to meet for an in-person interview. In Addis Ababa, participants chose a time and location for the interview that was convenient to them. The two local researchers in Tigray recruited participants at public health facilities with support from health facility staff. Health facility staff would approach potential participants prior to their abortion to enquire about their interest in participating. If they were interested, one of the local researchers would arrive at the health facility immediately after the client had received care to enroll them in the study. People were eligible to participate if they were 15 years or older, able to provide informed consent, able to speak Amharic or Tigrinya, and had had an abortion within 6 months prior to recruitment.

The interview guide was developed as a part of the larger aforementioned study and was based on quality of care and person-centered care frameworks (29, 37, 38) as well as prior studies on abortion quality with the goal of understanding people's experiences of and priorities in abortion care. The interview guide included questions on participants understanding of the local legal context and on understanding client-provider interactions. While the interview guide was similar across the four contexts, the local research team in Ethiopia helped edit the guide to ensure clarity and cultural applicability. The interviewers were fluent in either Amharic or Tigrinya and the interview guide was translated from English and reviewed for accuracy.

All participants gave verbal consent to participate and to be audio-recorded prior to the start of the interview. All interviews took place in a private location and lasted between 40 and 60 min. Interviews were audio-recorded, transcribed verbatim, and translated to English for analysis. EG conducted quality assurance checks on the transcripts to ensure accurate translation. Participants were compensated for their time and travel expenses which equated to approximately $5 USD. This study was approved by Allendale Investigational Review Board (IRB) based in the United States (Protocol Number: ASQ092018), MSI Reproductive Choices' Ethics Review Committee (Protocol Number: 023-18), and the IRB of St. Paul's Hospital Millennium Medical College in Addis Ababa (Reference Number: PM23/56).

An initial codebook was developed for the larger study, using themes from the interview guide, and was amended to include emergent themes. This codebook was applied to the interviews with Ethiopian abortion clients by CB and AK, who initially double coded two transcripts to ensure intercoder reliability. Coding was conducted with MAXQDA 2018 (VERBI Software, 2019). We conducted thematic analysis grouping together codes related to client-provider interactions. During this process, themes of denial and dissuasion arose and resulted in closer examination of negative interactions from participant's perspectives. We drafted additional analysis memos and created matrices of participant experiences with denial, disrespect, and dissuasion, as well as their understanding of the abortion law. As a form of data validation, EG reviewed the analysis and interpretation of the findings presented in this manuscript. Quotes have been translated into English; all participant names are pseudonyms.

## Results

This analysis included 23 semi-structured in-depth interviews, 11 conducted in Addis Ababa and 12 in Tigray from December 2018 to January 2019. The participants ranged in age from 19 to 38 years, with an average age of 24 years. Nearly half of participants had children (*n* = 11, 47.8%) and the majority of participants also reported being unmarried (*n* = 15, 65.2%). The vast majority of participants (*n* = 20, 87%) had a medication abortion at a health facility; only 3 participants (13%) had Manual Vacuum Aspiration (MVA) abortions. About two thirds of participants were in their first trimester (*n* = 16, 69.9%), while one third of participants were in their second trimester (*n* = 7, 30.4%). Four participants (17.4%) reported having had a prior abortion (See Table 1).

**Table 1:** Participant characteristics.

	*N* = 23
	*n* (%)
Age (years)	
Mean	24
Range	19–38
Had children	
Yes	11 (47.8%)
No	12 (52.2%)
Marital Status	
Married	7 (30.4%)
Unmarried	15 (65.2%)
Divorced	1 (4.3%)
Abortion method	
Medication abortion	20 (87%)
Manual Vacuum Aspiration (MVA)	3 (13%)
Gestational age	
≤12 weeks	16 (69.6%)
>12 weeks	7 (30.4%)
Prior abortion	
Yes	4 (17.4%)
No	19 (82.6%)

### Fear of being denied abortion services

Fear of being denied abortion services arose prominently as a theme, with participants frequently reporting that they experienced concerns about denial prior to and during abortion care for a variety of reasons. The fear of denial was compounded by participants' personal circumstances, for example, this participant who had a prior abortion was worried about stigma and denial, “*I was afraid they will ask me why I let this happen for the second time. I was afraid they will be judging me and even deny me the service because it is my second time” (Marta, Private Facility).* Similarly, a participant who had their abortion later in pregnancy was worried about how the providers would respond, “*I had fear and I thought that the health care providers may not allow me to abort it. Maybe they would ask me why I didn’t come sooner since the pregnancy was 5 months” (Shewit, Public Facility)*. Participants who were seeking an abortion based on legally permissible reasons also worried whether providers would deny them an abortion. Several participants who had been raped or victims of incest were afraid that providers would not believe them or would not provide them the services, Letay, who was raped, explains, “*When I came to the hospital, I was very worried … I was worried because it happened out of my marriage. And I was worried whether the health professionals will help me or not and if I would get the service or not” (Letay, Public Facility)*.

Participants' fear of denial may have been influenced by their lack of knowledge of their legal right to access abortion services. Participants' knowledge of abortion law in Ethiopia varied greatly with less than half of participants reporting any knowledge of the legal clauses. While some participants named numerous clauses under which they presumed abortion was legal, their knowledge was fragmented and often did not include the full scope of legally permissible reasons. Among those that knew there were legal clauses for abortion, most only mentioned rape and incest and no other clauses. Many participants believed that providers determined whether an abortion could be performed, “*It's the doctor's decision” (Frewoyni, Public Facility)*. Another participant explained in more detail,

*“If we are eligible, they tell us…What I know is the healthcare provider can’t do an abortion for everyone. They work based on documents or rules. They ask why we came and if the reason is acceptable, they give us the service.” (Birhan, Public Facility)*

Birhan recognized that there were some regulations around abortion services and identified the provider as the gatekeeper. At the same time, another participant described the advice they received from a friend,

*“I was stressed so I asked [a friend] about the health care providers, whether they are strict and what they will say if I go there seeking abortion, then she told me to say I got raped and I can’t raise it if the baby is born, after that the health care providers will not say anything other than providing you the service.” (Birkti, Public Facility)*

While the interaction that the participant describes acknowledges the power providers hold, it also demonstrates people's ingenuity in sharing information amongst themselves to subvert the power of gatekeepers and reduce their chances of being denied an abortion.

### Experiences of disrespect: denial and dissuasion

#### Denial along pathways to care

While all participants eventually succeeded in having an abortion, along their pathways to accessing care nearly half of participants experienced being denied services, sometimes at multiple points of care. Clients experienced denial of services for a variety of reasons including lack of financial resources, providers simply not wanting to provide the service, providers not believing their reason for seeking abortion, having had a prior abortion, seeking care later in pregnancy, moral judgement from providers and providers' perception of negative health implications related to an abortion. Many of these reasons aligned with the fears that participants shared about denial. One participant explained that they visited a facility multiple times, which was common among participants,

*“I didn’t have enough money then I told them I didn’t have money the girl at the reception told me she can’t do anything, I went back, to get money and come back […] and then I went back there after one month, I told them, “this thing happened, I didn’t have money at the time” and they were mean to me […] I came with money, was going there for a second time and they thought I didn’t have money this time too but I had more than enough money.” (Hawi, Private Facility)*

Being turned away from a facility, or multiple facilities, during the pathway to abortion care meant that participants could face judgement and condemnation from staff and providers at multiple points. The following participant notes the stigma they had to overcome to find a provider who was willing to provide an abortion,

*“When I came here they did not give me the service …They said they will not provide me the service, advised me not to abort, and sent me home. They told me I am killing a human being for the wrong reason, saying this is going to pass and I will be ok … but I didn’t listen and went to another facility to get the care. I was disappointed and frustrated. Then I went to private institution where they agreed to give me the service.” (Birhan, Public Facility)*

Many other participants also recounted experiences of providers serving as the moral arbiters of their abortions, pressuring people not to terminate their pregnancies. Other participants were denied care because of providers' erroneous perception of abortion being dangerous. For example, Selam was denied services and was told that having an abortion might end their life,

*“The doctors told me when I went to the health centers to consult. They say they don't have the service in their facility. They told me wherever I go the abortion will be done using an instrument and I may even end up dead during the procedure or I may come out alive. The doctor told me the death is because of severe bleeding. He told me even if I go to a private clinic, I may end up dead. Finally, I decided to have the abortion even if I risk death. I preferred it rather than telling my family.” (Selam, Private Facility)*

Participants faced misinformation and judgement from providers in their continued efforts to dissuade participants from having an abortion. However, even in the face of denial of care and fearmongering, participants persevered overcoming their fear of death to find the care they needed.

#### Dissuasion at the point of abortion care

Even after participants reached the facility where they would ultimately obtain care, they frequently reported providers attempting to dissuade them from having an abortion. Reflecting both the fears they had expressed and experiences of denial along their pathways to care, participants faced providers who required them to justify their decision to have an abortion, who showed disbelief in their personal circumstances, who attempted to convince them to keep the pregnancy, who spread misinformation about the safety of the procedure, and who morally disagreed with their decision. One participant shared how they felt the provider did not believe their reason for seeking an abortion,

*“I asked about the service and told the health care provider I wanted to terminate the pregnancy because I was raped. However, the healthcare providers didn’t trust me. They said that I was not raped, but was pregnant from my boyfriend, and told me to go and try to convince him [to keep the child] but I told them that it's not from my boyfriend. Then they understood and gave me the service.” (Abrehet, Public Facility)*

After Abrehet was able to obtain an abortion, this participant reflected on their initial interaction stating, “*I understand that all they said was for my sake and I felt happy for that,”* reflecting discordance between the recount of their experience of treatment and how they perceived this treatment. Similarly, many clients shared experiences of providers interrogating them and requesting that they justify their decision to have an abortion, “*You also have to go through so many investigations…They were repeatedly asking why I was getting an abortion in the doctor's room” (Adiam, Public Facility).* Adiam also shared discordance when reflecting on their experience of care, they stated that they never felt judged and had “*no negative interactions”* with any of the providers or staff.

Clients reported that after interrogating them for their decisions, providers then attempted to persuade them to keep the pregnancy. Frewoyni describes the provider's attempt to convince them to keep the pregnancy,

*“He also asked me, “Why are you aborting it?” and I said, “Because I am poor, I can’t raise the kid.” Then he said, “It's better if you keep it and did your best to raise him.” I said, “How can I? I can’t!” and he said, “If you want to abort it, you will sign for it.”” (Frewoyni, Public Facility)*

Frewoyni's provider also seemed to invoke a vague threat that Frewoyni would have to assume legal responsibility for their actions in his demand that they sign for their abortion. Participants also described how the providers or clinic staff attempted to dissuade them from obtaining an abortion by linking abortion to negative health outcomes, instilling fear in them and spreading misinformation. One participant explained how a provider made them fear for their life,

*“I was in a dilemma. I was thinking of getting the abortion but I was scared for my health and my life…When the health center health provider told me aborting a 5-month pregnancy could be dangerous for me, I was scared for my life.” (Genet, Public Facility)*

Rather than reassure Genet of the safety of the procedure, the provider seemingly used fear as a tactic to dissuade them from having an abortion. Participants also spoke about providers who questioned their decisions on moral grounds,

*“I talked to the doctor. He was trying to convince me not to terminate the pregnancy. He told me that abortion is not good. However, after I told him that I am not ready and don’t want it, he accepted my decision.” (Seada, Private Facility)*

As Seada describes, clients reported that providers tested their rationale and their necessity for an abortion before conceding and providing the service. Despite facing disrespect from providers, participants commonly felt that the clinic staff and providers were acting in their best interest, “*This is out of concern. They think maybe we will listen to them that way.”* Semira classified the condemnation received during their abortion as a positive aspect of care,

*“This shows you the service is good. If they do not condemn you, I may not feel good at all. My provider was an older man. When he condemned me, I felt like my father was speaking to me.” (Semira, Private Facility)*

Semira highlights a trend present amongst several participants who either felt that providers were expressing care in their condemnation or who felt they had received good service despite experiencing disrespect, denial, or dissuasion.

## Discussion

Across 23 in-depth interviews with clients from public and private health facilities in two distinct geographic locations in Ethiopia, we found that many participants experienced denial of abortion services along their pathway to care and attempts by providers to dissuade them prior to providing an abortion. Underlying both the denial and the dissuasion were reports of disrespect and condemnation from providers. Participants described how providers doubted or forced them to justify their reasons for having an abortion, stigmatized them for seeking multiple abortions or later abortions, and ascribed misinformation about abortion safety. Despite reports of denial, dissuasion and disrespect, abortion clients generally felt that providers had their best interest at heart and were grateful for having access to an abortion.

Participants faced providers who acted as gatekeepers to abortion services, denying and dissuading them even in cases where they explicitly gave legal reasons for seeking care. While these interviews were focused on clients' experiences of abortion, we know from research in Tunisia, Zambia and Zimbabwe that providers (whether pharmacists, front desk staff, or medical practitioners) often serve as gatekeepers to abortion access, navigating ambiguous or confusing laws and their own personal beliefs (39–41). Additionally, participants' limited knowledge of their legal right to abortion access may have meant that they were unable to advocate for themselves in situations where providers denied care or questioned their decision to have an abortion. This type of questioning can force people to relive traumatic events, such as rape or incest, by having to justify their reasons for wanting to have an abortion to providers. Eliminating client questioning would be a powerful step towards improving both person-centered care and overall quality of care. This study underlines the need for increased awareness of availability of abortion services within the legal limitations in Ethiopia, which could serve as a powerful tool for patient advocacy (42, 43). Beyond increasing awareness, there are opportunities for context-specific provider and administrator trainings and support to increase access to abortion services. Interventions could focus on human rights based frameworks (44), stigma-reduction workshops (45), and elements of person-centered care including shared decision-making and trust (29).

Abortion clients in this study reported examples of disrespect and abuse when they described being denied services they were legally eligible for, interrogation, explicit condemnation, and receiving erroneous information. Denial of care erodes trusting relationships with health systems and providers. Previous work has showed that trustworthy providers were a fundamental aspect of client-reported high-quality abortion care (25, 46, 47). Further, disrespect and abuse during maternal child care is rampant in patient-provider interactions (48–51), and abusive care, or obstetric violence, is now seen as a human-rights violation and a symptom of structural violence (48, 52, 53). A study in Ethiopia on disrespect and abuse during labor and delivery found that providers lacked training on respectful, responsive care, and on counseling (51). In addition to a lack of training on respectful client-provider interactions, providers denying and dissuading people from having an abortion might also be a greater symptom of social norms and abortion-related stigma in Ethiopia. Previous studies have shown the way that providers in Ethiopia describe the challenge of balancing their religious faith and values with their professional obligations to provide abortion (15). The internal conflict that some providers feel about providing abortion may perpetuate stigmatizing interactions with people seeking an abortion, and ultimately, may lead to poor quality of interpersonal care (54). In some cases people seeking abortions also share the same personal and religious beliefs about abortion as their providers (55) and, as such, may believe that they deserve the disrespectful and abusive care that they are receiving.

Among those that reported facing disrespectful care, participants still tended to describe their experiences and interactions as positive. This might be influenced by abortion stigma, fear, and expectations of care. The dissonance participants expressed between the type of treatment they received (judgement/negative) and their assessment of that same treatment (rationalization/positive) may be reflective of the same normative beliefs around abortion that influence provider's negative, judgmental treatment of abortion clients. It is common in abortion care broadly for people to report feeling satisfied with the services they receive and be grateful, regardless of the type of care, because they are no longer pregnant (56–59). This creates challenges for measuring quality of care of abortion services and, ultimately, for improving service delivery (60). A study in Ethiopia documenting women's satisfaction with facility-based maternal health care found similarly complex reflections on experiences of care with women largely reporting being satisfied if they had delivered a healthy child safely despite experiencing what might be considered abusive care (22). Our findings echo the complexities of basing quality of care measures on metrics like “satisfaction” and similarly highlight the importance of more nuanced client-centered indicators that take into consideration this type of discordance. In addition, monitoring and evaluation efforts at abortion facilities should take into account negative experiences of care in order to document their frequency and identify points of intervention.

As with all research, this study had its limitations. While our objective was to understand experiences of abortion care from client's perspectives, we recognize that conducting interviews with providers would have added richness and a deeper level of understanding of client-provider interactions. Further research is needed to understand how abortion providers and clinic staff describe their care and the role they play in determining eligibility for abortion services in Ethiopia. Second, we did not structure our analysis to make comparisons between experiences of clients in private vs. public facilities in the country, nor did we structure our analysis to draw conclusions on the unique geographic and contextual differences that might have influenced participant's experiences of abortion care. Third, while the participants in this study described their personal experiences of denial and dissuasion, ultimately, they all received abortions. Their experiences may not be representative of others who were not able to receive abortion care. Finally, this study was completed prior to great civil unrest that has since broken out in Ethiopia (61) and the findings cannot reflect how people's experiences accessing abortion have changed during this time. However, the lessons learned from this analysis could help improve legal access to abortion as the country heals from this internal conflict and seeks to rebuild access to health services – hopefully changing the way providers give abortion care and empowering people with knowledge of their rights so that everyone can have a high-quality abortion experience.

## Conclusion

Along the pathway to abortion care and during care, abortion clients in Ethiopia faced denial, dissuasion and disrespect from healthcare professionals contributing to the barriers they already face in accessing care and negatively impacting their experiences of care. Denial and dissuasion are both forms of disrespect that manifested in providers not believing clients, shaming them, and casting moral judgement on client's personal decisions and lives, and ultimately providers acting as gatekeepers to abortion services. Increasing awareness of abortion laws such that clients understand their rights and interventions aimed at addressing provider's beliefs and understanding of person-centered care could help reduce barriers to accessing care and improve the quality of abortion services. Furthermore, increasing efforts to understand and measure negative experiences of abortion care may help inform additional interventions to ultimately improve quality of care of abortion services for everyone.

## Data Availability

The dataset generated and analyzed during the study is not publicly available in order to maintain confidentiality and reduce risk to the participants. The interview guide is available from the corresponding author on reasonable request. Requests to access the interview guide should be directed to cbercu@ibisreproductivehealth.org.

## Footnotes

^1^We acknowledge that not all people who are capable of pregnancy and/or abortion identify as women. We use the term ‘women’ when referring to prior research that describes study subjects as such and we use the gender inclusive term ‘people’ whenever possible to acknowledge the diversity of people with uteri and the diversity of pregnancy and abortion experiences.
